# Rationale and design of WEBCARE: A randomized, controlled, web-based behavioral intervention trial in cardioverter-defibrillator patients to reduce anxiety and device concerns and enhance quality of life

**DOI:** 10.1186/1745-6215-10-120

**Published:** 2009-12-23

**Authors:** Susanne S Pedersen, Viola Spek, Dominic AMJ Theuns, Marco Alings, Pepijn van der Voort, Luc Jordaens, Pim Cuijpers, Johan Denollet, Krista C van den Broek

**Affiliations:** 1 CoRPS, Department of Medical Psychology, Tilburg University, Tilburg, the Netherlands; 2 Department of Cardiology, Thoraxcenter, Erasmus Medical Center, Rotterdam, the Netherlands; 3 Department of Cardiology, Amphia Hospital, Breda, the Netherlands; 4 Department of Cardiology, Catharina Hospital, Eindhoven, the Netherlands; 5 Department of Clinical Psychology, VU University Amsterdam, the Netherlands

## Abstract

**Background:**

The implantable cardioverter defibrillator (ICD) is generally well accepted, but 25-33% of patients experience clinical levels of anxiety, depression, and impaired quality of life (QoL) following implantation. Few trials in ICD patients have investigated whether behavioral intervention may mitigate the development of these adjustment problems. We present the rationale and study design of the **WEB**-based distress management program for implantable **CAR**dioverter d**E**fibrillator patients (WEBCARE) trial.

**Methods:**

WEBCARE is a multi-center, multi-disciplinary, randomized, controlled behavioral intervention trial designed to examine the effectiveness of a web-based approach in terms of reducing levels of anxiety and device concerns and enhancing QoL. Consecutive patients hospitalized for the implantation of an ICD will be approached for study participation while in hospital and randomized to the intervention arm (n = 175) versus usual care (n = 175) at baseline (5-10 days post implantation). Patients will complete assessments of patient-centered outcomes at baseline, 14, 26, and 52 weeks after implantation. Patients randomized to the intervention arm will receive a 12-week web-based behavioral intervention starting 2 weeks after implantation. Primary endpoints include (i_i_) patient-centered outcomes (i.e., anxiety, depression, ICD acceptance, QoL); (i_ii_) health care utilization; and (i_iii_) cost-effectiveness. All primary endpoints will be assessed with standardized and validated disease-specific or generic questionnaires. Secondary endpoints include (ii_i_) cortisol awakening response; and (ii_ii_) ventricular arrhythmias.

**Discussion:**

WEBCARE will show whether a behavioral intervention using a web-based approach is feasible and effective in reducing anxiety and ICD concerns and improving QoL in ICD patients.

**Trial Registration:**

http://www.ClinicalTrials.gov. Identifier: NCT00895700.

## Background

Implantable cardioverter defibrillator (ICD) therapy is considered the treatment of choice for the prevention of sudden cardiac death (SCD) both in patients who have survived life-threatening arrhythmias (secondary prevention) and in patients at risk for these arrhythmias due to coronary artery disease and left ventricular dysfunction (primary prevention) [1]. The number of ICD implantations has risen considerably, since the first ICD was implanted in humans [2-4]. This increase in implantation rates can in part be attributed to expansion of the indications for ICD implantation to also include primary prevention, due to ICD therapy being superior to anti-arrhythmic drugs in the prevention of SCD in these high-risk patients [5-7].

Despite the medical benefits of ICD therapy, and the device generally being well accepted by the majority of patients [8], 25-33% of patients experience clinical levels of anxiety, depression, and impaired quality of life (QoL) following implantation [9-12]. These difficulties may be attributed to actual ICD shocks [13,14], but also to device-related concerns including fear of shocks [15,16], ICD advisories (i.e., a notification from ICD manufacturers that the hardware may potentially malfunction) [17], and the psychological make-up of the patient, including lack of optimism [18] and personality factors, such as the *distressed *(Type D) personality [11,12,19]. The prevention of the manifestation of psychological distress in ICD patients is important, given preliminary evidence that distress may increase the risk of ventricular tachyarrhythmias [20-22]. Hypercortisolemia, reflecting a dysregulation of the hypothalamus-pituitary-adrenocortical axis, may provide one of the mechanisms linking psychological factors to ventricular tachyarrythmias due to increased inflammation arising from chronic stress [23].

Preliminary evidence suggests that ICD patients benefit from psychological and behavioral intervention, with the largest effect being found in reductions of symptoms of anxiety and improved exercise capacity [24]. One intervention trial has also found a reduction in cortisol levels [25]. However, the number of large-scale intervention trials in ICD patients is scarce [24,26], with the majority of these trials having used a nurse-based approach, cognitive behavioral therapy as stand-alone therapy, or in combination with cardiac rehabilitation. A web-based intervention may be equally effective and have advantages over these more traditional forms [27]. Advantages of a web-based intervention include its low-threshold accessibility via the internet, which makes it logistically feasible for most patients to participate, as they do not have to take time off work, but can access the intervention at home in their own time. In addition, a web-based approach safeguards the patient's anonymity, and the patient avoids the stigma associated with traditional, face-to-face therapy. Such an approach may be particularly useful for patients with a Type D personality profile, who are at increased risk of adverse health outcomes, as they do not share their emotions in social interactions due to fear of rejection [28].

## Design

The **WEB**-based distress management program for implantable **CAR**dioverter d**E**fibrillator patients (WEBCARE) is a Dutch multi-center, multi-disciplinary, randomized, controlled behavioral intervention trial designed to examine the effectiveness and feasibility of a web-based approach in terms of reducing levels of anxiety and device concerns and enhancing QoL in ICD patients. The trial has been registered on http://www.ClinicalTrials.gov (NCT00895700).

### Study population and eligibility criteria

Consecutive patients (N = 350) hospitalized for the implantation of an ICD in three medical centers in The Netherlands will be approached for study participation while in hospital and randomized to the intervention arm (n = 175) versus usual care (n = 175) at baseline (5-10 days post implantation). Patients (i) being implanted with an ICD, (ii) between 18-75 years of age, (iii) speaking and understanding Dutch, (iv) with access to and ability to use the internet, and (v) providing written informed consent will be eligible to participate. Patients with a life expectancy less than 1 year, with a history of psychiatric illness other than affective/anxiety disorders, on the waiting list for heart transplantation, or with insufficient knowledge of the Dutch language will be excluded.

### Study procedure, randomization and follow-up

The study protocol will be approved by the medical ethics committees of the participating centers. The study will be conducted according to the Declaration of Helsinki, as amended in 2008 by the World Medical Association, and all patients will be informed orally and in writing about the purpose, rights, and possible benefits/risks of the study.

**Figure 1:**
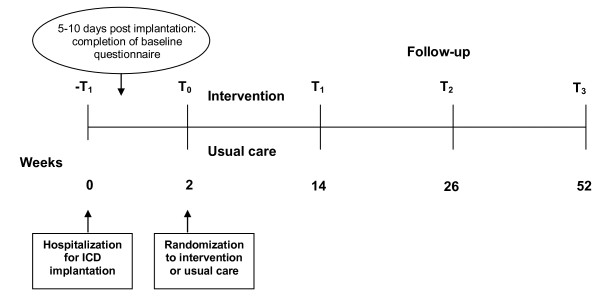
**Schematic representation of the study procedure**.

Patients will be invited to participate in the study while hospitalized for their ICD implantation. Patients responding positively to the invitation will be given the informed consent form and the baseline questionnaire package. In order to avoid measuring pre-operative stress, patients will be asked to complete the baseline assessment between 5 and 10 days (T_0_) after implantation. If the baseline questionnaire is not returned within 7 days, patients will receive a reminder telephone call. When the signed informed consent form and completed baseline assessments are received at the trial coordinating center, the coordinating center will randomize the patient either to the treatment arm or to usual care. There will be a separate randomization list for each participating hospital. The patient will be informed by telephone to which condition he/she has been randomized and the procedure for the rest of the study. If the patient is randomized to the internet intervention, he/she will receive a login and password in order to be able to access the intervention. The day of randomization will be the first day of the intervention period, with the duration of the intervention being 12 weeks. For both study conditions, the follow-up assessments will take place 14 (T_1_), 26 (T_2_), and 52 weeks (T_3_) post implantation. All randomized patients, irrespective of condition, will be followed until the scheduled study end. A schematic representation of the study design is shown in Figure 1.

### Study endpoints

Primary endpoints include (i_i_) patient-centered outcomes (i.e., anxiety, depression, ICD acceptance, QoL); (i_ii_) health care utilization; and (i_iii_) cost-effectiveness of the intervention. Secondary endpoints include (ii_i_) cortisol awakening response; and (ii_ii_) ventricular arrhythmias.

#### Assessment of primary endpoints

All primary endpoints in addition to psychological factors, such as Type D personality [29], will be assessed with standardized and validated measures shown to have acceptable psychometric properties (Table 1). Disease-specific measures include the Florida Shock Anxiety Scale [30], the Minnesota Living with Heart Failure questionnaire [31], the ICD Patient Concerns questionnaire [16], and the Florida Patient Acceptance Survey [32,33].

**Table 1:** Patient-centered outcomes and psychological factors assessed in the trial

Construct	Questionnaire		T_0_	T_1_	T_2_	T_3_
Anxiety	STAI-S	State Trait Anxiety Inventory (state only)	**x**	**x**	**x**	**x**
	FSAS	Florida Shock Anxiety Scale	**x**	**x**	**x**	**x**
	PDS	Posttraumatic Stress Scale		**x**	**x**	**x**
	DAI-4	Denollet Anxiety Inventory	**x**	**x**	**x**	**x**
Depressive symptoms	HADS	Hospital Anxiety and Depression Scale	**x**	**x**	**x**	**x**
	PHQ-9	Patient Health Questionnaire	**x**	**x**	**x**	**x**
Quality of life	SF-12	Short Form Health Survey 12	**x**	**x**	**x**	**x**
	MLWFQ	Minnesota Living With Heart Failure Questionnaire	**x**	**x**	**x**	**x**
ICD concerns	ICDC	ICD Patient Concerns Questionnaire	**x**	**x**	**x**	**x**
ICD acceptance	FPAS	Florida Patient Acceptance Survey	**x**	**x**	**x**	**x**
Health care utilization and cost-effectiveness	TiC-P	Trimbos/iMTA questionnaire for Costs associated with Psychiatric Illness	**x**			**x**
Type D personality	DS14	Type D Scale	**x**	**x**	**x**	**x**

**T**_0 _= Baseline; **T**_1 _= 14 weeks; **T**_2 _= 26 weeks; **T**_3 _= 52 weeks.

#### Assessment of secondary endpoints

Salivary cortisol will be assessed, using the Salivette (manufactured by Sarstedt, Etten-Leur, The Netherlands), at 3 out of the 4 time points, corresponding to the assessment of patient-centered outcomes and psychological variables (i.e., T_0 _= 5 to 10 days after ICD implantation; T_1 _= 14 weeks post-implantation; T_3 _= 52 weeks post-implantation). Due to test-retest reliability characteristics of cortisol [34,35], samples will be taken on two consecutive weekdays at each time point. Four samples will be taken at each time point and on the consecutive day, that is (a) when waking up, (b) 1/2 hour later, (c) 11.00 a.m., and (d) 3.00 p.m. Samples will be sent to the coordinating trial center, where they will be stored at -20°Celsius until assayed.

Information on ventricular arrhythmias will be gathered from the electrograms stored by the ICD that will be reviewed and classified by two experienced electrophysiologists from the EP staff of the participating centers, who are blind to whether patients are assigned to the treatment or usual care arm. In case the two reviewers disagree about the diagnosis, a third one will be consulted to reach a consensus. For each episode, the date, type, and mean cycle length of the tachyarrhythmia will be recorded, as well as the type and outcome of delivered ICD therapy. The arrhythmia will be classified as (1) ventricular tachyarrhythmia or (2) atrial tachyarrhythmia without a coexistent ventricular arrhythmia. Therapy triggered by ventricular tachyarrhythmia will be considered as appropriate, while therapy delivered for atrial tachyarrhythmia (including atrial fibrillation, atrial flutter, atrial tachycardia, sinus tachycardia) or T wave oversensing and noise will be defined as inappropriate

### Intervention versus usual care

Patients randomized to the treatment arm will receive an internet-based intervention starting 2 weeks after implantation. They will receive a username and password to access the intervention via the web. Every week an automated email will be sent to the participants to explain the contents and exercises for the coming week. All the information, as well as the exercise forms, will be downloadable from the website in case participants prefer to read the information on paper. Master's level psychology students, trained and supervised by psychologists will provide feedback on the completed exercises. This feedback is not meant to be therapeutic; it will be directed toward mastering the problem-solving strategies. The maximum amount of time the psychology students should spend on feedback will be 60 minutes per participant. The components of the intervention are presented in Table 2. The intervention has to be completed within 12 weeks, as we learnt in an earlier trial that one session a week is too fast a pace for most people. A typical session takes about 30 minutes and consists of an introduction, a discussion of the previous lesson's homework, new theory and homework for the subsequent week. The sessions are designed to be followed on a weekly or two-weekly basis. These time indications may vary among users. Besides the sessions, the participants have several resources at their disposal: a homework station, extra information, reading tips, useful links, and addresses for additional help. Patients randomized to the usual care arm will receive care as it is standardly offered to ICD patients in the three centers, comprised of an information booklet about ICD treatment, standard clinical follow-up visits, and home monitoring of the device (if applicable).

**Table 2:** Components of the 12-week web-based intervention

*Components*	*Topics dealt with*
**▪ Psycho-education about the ICD**	▪ Emotional reactions to ICD therapy
**▪ Problem-solving skills**	▪ Which aspects of ICD therapy may lead to distress
**▪ Cognitive restructuring**	▪ How to deal with shocks
	▪ Disease-specific issues and fears
**▪ Relaxation training**	▪ How to prevent the avoidance of activities
**▪ Personalized feedback by a therapist via the computer**	▪ Interpretation of bodily symptoms
	▪ How to cope with uncertainty
	▪ Help-seeking behaviour
	▪ How to cope with stress

### Statistical analysis and power calculation

The trial is designed to show whether a web-based behavioral intervention is effective in improving patient-centered outcomes compared to usual care. The power analysis was performed in relation to anxiety as the primary endpoint, since anxiety is a pertinent outcome measure in ICD patients due to the unique feature of the ICD being able to provide a shock. With (i) an expected between group effect size of .30., based on a recent meta-analysis of web-based intervention for symptoms of anxiety and depression [36] and taking into consideration that our population consists partly of patients with subclinical anxiety and depression levels; (ii) alpha = 0.05; (iii) power = 0.80 (two-sided test), 350 patients are needed (i.e., 175 in each condition). Although we had a response rate of 82% in an earlier study [16,19], based on a more conservative response rate of 50%, 700 patients need to be approached.

The effectiveness of the intervention will be examined using the intention-to-treat principle, with the inclusion of all randomized participants in the statistical analysis regardless of whether they completed the intervention or the follow-up measurements. Missing data will be imputed using regression imputation techniques. Univariable and multivariable regression analyses (both linear and Cox proportional hazard regression analysis) and analysis of variance and analysis of covariance with repeated measures will be used to investigate the effect of the intervention on the endpoints. The type of regression analysis will depend on the endpoint in question. Demographic, clinical and psychological variables will be included in multivariable analyses in order to control for their potential confounding effects. A p-value < 0.05 will be used to indicate statistical significance. All data will be analyzed using SPSS 17.0 for Windows (SPSS Inc., Chicago, Illinois).

## Discussion

The ICD is generally well accepted by patients [8], but 25-33% of patients experience clinical levels of anxiety, depression, and impaired QoL following implantation [9-12].

Preliminary evidence suggests that psychological and behavioral intervention may mitigate the development of adjustment problems in ICD patients, but the number of large-scale intervention trials in ICD patients is scarce [25-27]. In addition, all of these trials have used a nurse-based approach, cognitive behavioral therapy as stand-alone therapy, or in combination with cardiac rehabilitation. A web-based intervention may be equally effective and have advantages over these more traditional forms [28]. The **WEB**-based distress management program for implantable **CAR**dioverter d**E**fibrillator patients (WEBCARE) is a multi-center, multi-disciplinary, randomized, controlled behavioral intervention trial designed to examine whether a web-based approach leads to better patient-centered outcomes, in terms of reduced levels of anxiety and device concerns and improved QoL. The trial aims to include 350 patients from three Dutch centers.

## Abbreviations

ICD: Implantable cardioverter defibrillator; QoL: Quality of life; SCD: Sudden cardiac death.

## Competing interests

The authors declare that they have no competing interests.

## Authors' contributions

SSP in collaboration with VS, JD and KCB designed the study. PC provided the basis for the intervention, which was adapted by SSP, VS and KCB for ICD patients. SSP drafted the manuscript. VS, DT, MA, PV, LJ, PC, JD and KCB revised the manuscript critically. All have given their final approval of the version to be published.

## References

[B1] Zipes DP Camm AJ Borggrefe M Buxton AE Chaitman B Fromer M Gregoratos G Klein G Moss AJ Myerburg RJ Priori SG Quinones MA Roden DM Silka MJ Tracy C Smith SC Jr Jacobs AK Adams CD Antman EM Anderson JL Hunt SA Halperin JL Nishimura R Ornato JP Page RL Riegel B Blanc JJ Budaj A Dean V Deckers JW Despres C Dickstein K Lekakis J McGregor K Metra M Morais J Osterspey A Tamargo JL Zamorano JL American College of Cardiology/American Heart Association Task Force; European Society of Cardiology Committee for Practice Guidelines; European Heart Rhythm Association; Heart Rhythm Society ACC/AHA/ESC 2006 Guidelines for Management of Patients With Ventricular Arrhythmias and the Prevention of Sudden Cardiac Death: a report of the American College of Cardiology/American Heart Association Task Force and the European Society of Cardiology Committee for Practice Guidelines (writing committee to develop Guidelines for Management of Patients With Ventricular Arrhythmias and the Prevention of Sudden Cardiac Death): developed in collaboration with the European Heart Rhythm Association and the Heart Rhythm Society Circulation 2006 114 e385 484 10.1161/CIRCULATIONAHA.106.178233 16935995

[B2] Crespo EM Kim J Selzman KA The use of implantable cardioverter defibrillators for the prevention of sudden cardiac death: a review of the evidence and implications Am J Med Sci 2005 329 238 246 10.1097/00000441-200505000-00005 15894866

[B3] Seidl K Senges J Worldwide utilization of implantable cardioverter/defibrillators now and in the future Card Electrophysiol Rev 2003 7 5 13 10.1023/A:1023603118860 12766509

[B4] Lloyd-Jones D Adams R Carnethon M De Simone G Ferguson TB Flegal K Ford E Furie K Go A Greenlund K Haase N Hailpern S Ho M Howard V Kissela B Kittner S Lackland D Lisabeth L Marelli A McDermott M Meigs J Mozaffarian D Nichol G O'Donnell C Roger V Rosamond W Sacco R Sorlie P Stafford R Steinberger J Thom T Wasserthiel-Smoller S Wong N Wylie-Rosett J Hong Y American Heart Association Statistics Committee and Stroke Statistics Subcommittee Heart disease and stroke statistics -- 2009 update: a report from the American Heart Association Statistics Committee and Stroke Statistics Subcommittee Circulation 2009 119 e21 e181 10.1161/CIRCULATIONAHA.108.191261 19075105

[B5] Moss AJ Zareba W Hall WJ Klein H Wilber DJ Cannom DS Daubert JP Higgins SL Brown MW Andrews ML Prophylactic implantation of a defibrillator in patients with myocardial infarction and reduced ejection fraction N Engl J Med 2002 346 877 883 10.1056/NEJMoa013474 11907286

[B6] Bristow MR Saxon LA Boehmer J Krueger S Kass DA De Marco T Carson P DiCarlo L DeMets D White BG DeVries DW Feldman AM Comparison of Medical Therapy, Pacing, and Defibrillation in Heart Failure (COMPANION) Investigators Cardiac-resynchronization therapy with or without an implantable defibrillator in advanced chronic heart failure N Engl J Med 2004 350 2140 2150 10.1056/NEJMoa032423 15152059

[B7] Bardy GH Lee KL Mark DB Poole JE Packer DL Boineau R Domanski M Troutman C Anderson J Johnson G McNulty SE Clapp-Channing N Davidson-Ray LD Fraulo ES Fishbein DP Luceri RM Ip JH Amiodarone or an implantable cardioverter-defibrillator for congestive heart failure N Engl J Med 2005 352 225 237 10.1056/NEJMoa043399 15659722

[B8] Johansen JB Pedersen SS Spindler H Andersen K Nielsen JC Mortensen PT Symptomatic heart failure is the most important clinical correlate of impaired quality of life, anxiety, and depression in implantable cardioverter-defibrillator patients: a single-centre, cross-sectional study in 610 patients Europace 2008 10 545 551 10.1093/europace/eun073 18378633

[B9] Bostwick JM Sola CL An updated review of implantable cardioverter/defibrillators, induced anxiety, and quality of life Psychiatr Clin North Am 2007 30 677 688 10.1016/j.psc.2007.07.002 17938040

[B10] Matchett M Sears SF Hazelton G Kirian K Wilson E Nekkanti R The implantable cardioverter defibrillator: its history, current psychological impact and future Expert Rev Med Devices 2009 6 43 50 10.1586/17434440.6.1.43 19105779

[B11] Broek KC Van den Nyklicek I Van der Voort PH Alings M Denollet J Shocks, personality, and anxiety in patients with an implantable defibrillator Pacing Clin Electrophysiol 2008 31 850 857 10.1111/j.1540-8159.2008.01099.x 18684282

[B12] Pedersen SS Theuns DA Muskens-Heemskerk A Erdman RA Jordaens L Type D personality but not implantable cardioverter-defibrillator indication is associated with impaired health-related quality of life 3 months post-implantation Europace 2007 9 675 680 10.1093/europace/eum041 17434891

[B13] Schron EB Exner DV Yao Q Jenkins LS Steinberg JS Cook JR Kutalek SP Friedman PL Bubien RS Page RL Powell J Quality of life in the antiarrhythmics versus implantable defibrillators trial: impact of therapy and influence of adverse symptoms and defibrillator shocks Circulation 2002 105 589 594 10.1161/hc0502.103330 11827924

[B14] Sears SF Jr Conti JB Quality of life and psychological functioning of ICD patients Heart 2002 87 488 493 10.1136/heart.87.5.488 11997430 PMC1767118

[B15] Pedersen SS Theuns DA Erdman RA Jordaens L Clustering of device-related concerns and type D personality predicts increased distress in ICD patients independent of shocks Pacing Clin Electrophysiol 2008 31 20 27 10.1111/j.1540-8159.2008.01158.x 18181906

[B16] Pedersen SS van Domburg RT Theuns DA Jordaens L Erdman RA Concerns about the implantable cardioverter defibrillator: a determinant of anxiety and depressive symptoms independent of experienced shocks Am Heart J 2005 149 664 669 10.1016/j.ahj.2004.06.031 15990750

[B17] Broek KC Van den Denollet J Nyklicek I Voort PH Van der Psychological reaction to potential malfunctioning of implantable defibrillators Pacing Clin Electrophysiol 2006 29 953 956 10.1111/j.1540-8159.2006.00468.x 16981918

[B18] Sears SF Lewis TS Kuhl EA Conti JB Predictors of quality of life in patients with implantable cardioverter defibrillators Psychosomatics 2005 46 451 457 10.1176/appi.psy.46.5.451 16145190

[B19] Pedersen SS van Domburg RT Theuns DA Jordaens L Erdman RA Type D personality is associated with increased anxiety and depressive symptoms in patients with an implantable cardioverter defibrillator and their partners Psychosom Med 2004 66 714 719 10.1097/01.psy.0000132874.52202.21 15385696

[B20] Whang W Albert CM Sears SF Jr Lampert R Conti JB Wang PJ Singh JP Ruskin JN Muller JE Mittleman MA TOVA Study Investigators Depression as a predictor for appropriate shocks among patients with implantable cardioverter-defibrillators: results from the Triggers of Ventricular Arrhythmias (TOVA) study J Am Coll Cardiol 2005 45 1090 1095 10.1016/j.jacc.2004.12.053 15808769

[B21] Dunbar SB Kimble LP Jenkins LS Hawthorne M Dudley W Slemmons M Langberg JJ Association of mood disturbance and arrhythmia events in patients after cardioverter defibrillator implantation Depress Anxiety 1999 9 163 168 10.1002/(SICI)1520-6394(1999)9:4<163::AID-DA3>3.0.CO;2-B 10431681

[B22] Lampert R Joska T Burg MM Batsford WP McPherson CA Jain D Emotional and physical precipitants of ventricular arrhythmia Circulation 2002 106 1800 1805 10.1161/01.CIR.0000031733.51374.C1 12356633

[B23] Weber CS Thayer JF Rudat M Sharma AM Perschel FH Buchholz K Deter HC Salt-sensitive men show reduced heart rate variability, lower norepinephrine and enhanced cortisol during mental stress J Hum Hypertens 2008 22 423 431 10.1038/jhh.2008.11 18337758

[B24] Pedersen SS Broek KC van den Sears SF Jr Psychological intervention following implantation of an implantable defibrillator: a review and future recommendations Pacing Clin Electrophysiol 2007 30 1546 1554 18070312 10.1111/j.1540-8159.2007.00905.x

[B25] Sears SF Sowell LD Kuhl EA Kovacs AH Serber ER Handberg E Kneipp SM Zineh I Conti JB The ICD shock and stress management program: a randomized trial of psychosocial treatment to optimize quality of life in ICD patients Pacing Clin Electrophysiol 2007 30 858 864 10.1111/j.1540-8159.2007.00773.x 17584267

[B26] Lewin RJ Coulton S Frizelle DJ Kaye G Cox H A brief cognitive behavioural preimplantation and rehabilitation programme for patients receiving an implantable cardioverter-defibrillator improves physical health and reduces psychological morbidity and unplanned readmissions Heart 2009 95 63 69 10.1136/hrt.2007.129890 18070951

[B27] Kuhl EA Sears SF Vazques LD Conti JB Patient-assisted computerized education for recipients of implantable cardioverter defibrillators: a randomized controlled trial of the PACER program J Cardiovasc Nurs 2009 24 225 231 19390340 10.1097/JCN.0b013e31819c143d

[B28] Pedersen SS Denollet J Is Type D personality here to stay? Emerging evidence across cardiovascular disease patient groups Curr Cardiol Rev 2006 2 205 213 10.2174/157340306778019441

[B29] Denollet J DS14: standard assessment of negative affectivity, social inhibition, and Type D personality Psychosom Med 2005 67 89 97 10.1097/01.psy.0000149256.81953.49 15673629

[B30] Kuhl EA Dixit NK Walker RL Conti JB Sears SF Measurement of patient fears about implantable cardioverter defibrillator shock: an initial evaluation of the Florida Shock Anxiety Scale Pacing Clin Electrophysiol 2006 29 614 618 10.1111/j.1540-8159.2006.00408.x 16784427

[B31] Rector TS Kubo SH Cohn JN Patient's self-assessment of their congestive heart failure. Content, reliability, and validity of a new measure: the Minnesota Living with Heart Failure Questionnaire Heart Failure 1987 10 198 209

[B32] Burns JL Serber ER Keim S Sears SF Measuring patient acceptance of implantable cardiac device therapy: initial psychometric investigation of the Florida Patient Acceptance Survey J Cardiovasc Electrophysiol 2005 16 384 390 15828880 10.1046/j.1540-8167.2005.40134.x

[B33] Pedersen SS Spindler H Johansen JB Mortensen PT Sears SF Correlates of patient acceptance of the cardioverter defibrillator: cross-validation of the Florida Patient Acceptance Survey in Danish patients Pacing Clin Electrophysiol 2008 31 1168 1177 10.1111/j.1540-8159.2008.01158.x 18834469

[B34] Hellhammer J Fries E Schweisthal OW Schlotz W Stone AA Hagemann D Several daily measurements are necessary to reliably assess the cortisol rise after awakening: state- and trait components Psychoneuroendocrinology 2007 32 80 86 10.1016/j.psyneuen.2006.10.005 17127010

[B35] Kraemer HC Giese-Davis J Yutsis M O'Hara R Neri E Gallagher-Thompson D Taylor CB Spiegel D Design decisions to optimize reliability of daytime cortisol slopes in an older population Am J Geriatr Psychiatry 2006 14 325 333 10.1097/01.JGP.0000201816.26786.5b 16582041

[B36] Spek V Cuijpers P Nyklicek I Riper H Keyzer J Pop V Internet-based cognitive behaviour therapy for symptoms of depression and anxiety: a meta-analysis Psychol Med 2007 37 319 328 10.1017/S0033291706008944 17112400

